# Using fMRI and machine learning to predict symptom improvement following cognitive behavioural therapy for psychosis

**DOI:** 10.1016/j.nicl.2018.10.011

**Published:** 2018-10-10

**Authors:** Eva Tolmeijer, Veena Kumari, Emmanuelle Peters, Steven C.R. Williams, Liam Mason

**Affiliations:** a Department of Psychology, King's College London, Institute of Psychiatry, Psychology and Neuroscience, London, UK; b Centre for Cognitive Neuroscience, College of Life and Health Sciences, Brunel University London, Uxbridge, UK; c PICuP (Psychological Interventions Clinic for outpatients with Psychosis), South London and Maudsley NHS Foundation Trust, UK; d Department of Neuroimaging, King's College London, Institute of Psychiatry, Psychology and Neuroscience, London, UK; e University College London, Research Department of Clinical, Educational and Health Psychology, London, UK; f University College London, Max Planck Centre for Computational Psychiatry and Ageing Research, London UK

**Keywords:** Schizophrenia, Cognitive behavioural therapy, Affective processing, Neuroimaging, Positive psychotic symptoms, Depressive symptoms

## Abstract

Cognitive behavioural therapy for psychosis (CBTp) involves helping patients to understand and reframe threatening appraisals of their psychotic experiences to reduce distress and increase functioning. Whilst CBTp is effective for many, it is not effective for all patients and the factors predicting a good outcome remain poorly understood. Machine learning is a powerful approach that allows new predictors to be identified in a data-driven way, which can inform understanding of the mechanisms underlying therapeutic interventions, and ultimately make predictions about symptom improvement at the individual patient level. Thirty-eight patients with a diagnosis of schizophrenia completed a social affect task during functional MRI. Multivariate pattern analysis assessed whether treatment response in those receiving CBTp (n = 22) could be predicted by pre-therapy neural responses to facial affect that was either threat-related (ambiguous ‘neutral’ faces perceived as threatening in psychosis, in addition to angry and fearful faces) or prosocial (happy faces). The models predicted improvement in psychotic (*r* = 0.63, *p* = 0.003) and affective (*r* = 0.31, *p* = 0.05) symptoms following CBTp, but not in the treatment-as-usual group (n = 16). Psychotic symptom improvement was predicted by neural responses to threat-related affect across sensorimotor and frontal-limbic regions, whereas affective symptom improvement was predicted by neural responses to fearful faces only as well as prosocial affect across sensorimotor and frontal regions. These findings suggest that CBTp most likely improves psychotic and affective symptoms in those endorsing more threatening appraisals and mood-congruent processing biases, respectively, which are explored and reframed as part of the therapy. This study improves our understanding of the neurobiology of treatment response and provides a foundation that will hopefully lead to greater precision and tailoring of the interventions offered to patients.

## Highlights

• Machine learning models using neuroimaging data can predict response to CBTp.
• Neural responses to social threat predicted improvement in psychotic symptoms.
• Activation related to different social stimuli predicted distinct symptom domains.
• Predictors included activity in the hippocampus, frontal, and sensorimotor regions.

## 1 Introduction

The functional neurobiological underpinning of positive psychotic and affective symptoms in schizophrenia has been extensively studied (Birur et al., 2017; Karlsgodt et al., 2010; Henseler et al., 2010; Rolland et al., 2014; Northoff and Duncan, 2016; Sorg et al., 2013; Rotarska-Jagiela et al., 2009; Skelly et al., 2008; Kumari et al., 2016) and there is considerable promise in using these neurobiological markers to improve the precision of interventions at the individual patient level (Woo et al., 2017). To date, four studies have examined the neural predictors of cognitive behavioural therapy for psychosis (CBTp) treatment outcomes (Kumari et al., 2010; Kumari et al., 2009; Premkumar et al., 2009; Premkumar et al., 2015), the primary psychological intervention for this patient group (National Collaborating Centre for Mental Health, 2009). However, none of these studies used machine learning methods to investigate neural predictors at the individual patient level. Currently, only three studies have made successful predictions about individual CBT treatment outcomes using such analytical approaches, none of which was in schizophrenia patients (Månsson et al., 2015; Klumpp et al., 2017; Reggente et al., 2018). However, individual predictions are an important step towards better tailoring of treatment by utilising patient-specific markers to provide an objective estimate of treatment outcomes. Incorporation of neural predictors into existing assessment procedures can inform clinical decision-making regarding the use of additional psychological therapies to improve treatment effectiveness (Drake et al., 2014) and the resources that support CBTp. This is crucial considering that only approximately 50% of patients may experience clinically significant improvement following CBTp (Wykes et al., 2008) and limited clinical service resources mean that, even in the UK where it is a NICE (National Institute for Health and Care Excellence (NICE), 2014)-recommended treatment, only approximately 10% of patients receive therapy in routine services (Schizophrenia Commission, 2012).

Supervised machine learning methods, such as multivariate pattern analysis, are a powerful tool for identifying the neural predictors of treatment response since the analysis involves building an algorithm that can make predictions at the individual patient level (Orrù et al., 2012). This data-driven approach can detect subtle patterns of distributed activity predicting clinical outcomes (Orrù et al., 2012), such as treatment outcomes or disorder course. Brain responses to clinically relevant stimuli may be most likely to yield meaningful predictions about symptom improvement. A highly replicated finding in psychosis is a bias towards perceiving facial expressions as threatening, including evidence for the misattribution of threat to neutral facial expressions (Potvin et al., 2016; Underwood et al., 2015), which has been put forward as one route to paranoia (Underwood et al., 2015; Green and Phillips, 2004; Underwood et al., 2016a). Neuroimaging markers for this bias include elevated responses in a number of regions involved in threat perception and emotion processing (Potvin et al., 2016; Taylor et al., 2012; Hall et al., 2008). This network includes a number of limbic regions, including the amygdala, hippocampus and insula, as well as visual and motor areas (Taylor et al., 2012; Delvecchio et al., 2012; Li et al., 2010). The amygdala and insula are part of the ventral network which includes the anterior cingulate and the ventrolateral prefrontal cortex (PFC), whereas the hippocampus belongs to the dorsal system which includes the dorsolateral and dorsomedial PFC (Underwood et al., 2015). The latter is involved in the regulation of emotions whereas the former is important for threat appraisal (Underwood et al., 2015; Phillips et al., 2003). Sensorimotor regions, together with frontal regions, are crucial for the development of behavioural responses in a context-dependent matter (Janak and Tye, 2015). Neuroimaging studies on affective face processing have reported reduced activation in frontal areas, but increased activation in threat and sensorimotor regions in patients with psychosis in comparison to healthy controls (Taylor et al., 2012; Hall et al., 2008). In particular, the misattribution of threat to neutral stimuli has been associated with increased activation in the precentral and postcentral gyrus as well as the parietal lobule (Habel et al., 2010). Elevated threat-related activity in response to neutral facial expressions suggests they are not neutral in psychosis, which seems consistent with the interpretation of a heightened tendency to perceive threat when facial expressions are ambiguous (Potvin et al., 2016; Underwood et al., 2015; Underwood et al., 2016a). The evidence for increased activation for both ambiguous and threatening affect suggests the facial affect task is highly sensitive to aberrant threat and salience processing in psychosis. Additionally, evidence for reduced brain activation responses to happy facial expressions in major depression (Groenewold et al., 2013; Stuhrmann et al., 2011) suggests the task is sensitive to altered processing of prosocial affect in those who experience depressive symptoms. Moreover, differences in task activation have been shown to relate to both the type and severity of symptoms (Gur et al., 2007; Michalopoulou et al., 2008), making the facial affect task a potential assay for predicting post-therapy improvement in psychotic and affective symptoms.

CBTp is an effective intervention for psychosis (Bighelli et al., 2018) and is accompanied by improvements in social, occupational, and psychological functioning (Wykes et al., 2008; Pfammatter et al., 2006). The primary aim of CBTp is helping patients to understand and reframe threatening appraisals of their psychotic experiences, become less distressed and live a personally meaningful life (Birchwood and Trower, 2012; Garety and Hardy, 2017). Affective symptoms are frequently reported in psychosis (American Psychiatric Association, 2013) and are a common target of therapy by emphasising techniques that help patients recognise and change mood-congruent biases and unhelpful thinking styles (Birchwood and Trower, 2012; Kuipers et al., 2006), with evidence of reduced depression following CBTp (Garety et al., 2008; Peters et al., 2010). One neural mechanism proposed to underlie treatment effects in CBTp involves an increased recruitment of higher-order brain networks to regulate brain regions involved in threat and salience perception (Mason et al., 2016). This has been probed experimentally using the facial affect processing task, which has shown that improvement in positive psychotic symptoms (Mason et al., 2016; Kumari et al., 2011) and depressive symptoms (Mason et al., 2016) correlates with changes in functional neurobiology (Mason et al., 2016; Kumari et al., 2011; Mason et al., 2017). In particular, CBTp-led reductions in activation of the threat network in response to affective stimuli (Kumari et al., 2011) and concomitant increases in connectivity between the left amygdala and inferior parietal lobule correlated with improvement in positive symptoms of psychosis (Mason et al., 2016). These post-CBTp connectivity changes between the amygdala and the inferior parietal lobule as well as the dorsolateral PFC have also been found to be predictive of long-term affective and psychotic symptoms across several years (Mason et al., 2017). Whilst these studies have provided insight into the neural mechanisms underlying therapeutic change, they have not provided neural predictors of treatment outcomes for individual patients. The dorsolateral PFC might be a particularly promising region, since increased activity in this area in response to a working memory task predicted good outcomes following CBTp (Kumari et al., 2009). Additionally, activation in networks including the amygdala and parietal lobule, which have been implicated in the neural mechanism of CBTp, have been found to predict individual response to CBT for social anxiety disorder (Månsson et al., 2015). However, no study to date has examined whether pre-therapy neural responses to facial affect can be used to make individual predictions about CBTp outcomes.

The present study used the dataset of a case-controlled study that previously found that CBTp led to significant changes in the functional neurobiology of social threat processing, compared to treatment-as-usual (Kumari et al., 2011). Here, we employ multivariate pattern analysis to identify predictors of response to CBTp from pre-therapy functional MRI. We employed a two-step methodology. The first step involved identification of potentially predictive regions and the second step involved formation of a predictive model that utilised region-specific activation patterns to make a function that can make predictions at the individual patient level. We examined all available forms of facial affect processing, allowing the machine learning model to identify the specificity of the activation patterns for each facial condition for predicting treatment response in different symptom domains. We hypothesised that improvement in positive psychotic symptoms and depressive symptoms would be uniquely predicted by neural responses to threat-related (including ambiguous ‘neutral’ faces in addition to angry and fearful faces) and prosocial affect (happy faces), respectively. Caveated by the data-driven approach and the absence of studies investigating predictors of CBTp outcomes from social affective neural processing, we hypothesised that the dorsolateral PFC and the amygdala, suggested to be implicated in the neurobiological mechanism underlying CBTp (Mason et al., 2016), would predict post-CBTp improvement in both positive psychotic and affective symptoms.

## 2 Materials & methods

### 2.1 Participants & design

Participants were 38 patients who received either treatment-as-usual (TAU group) or CBTp on top of their regular care (+CBTp group), in a case-control cohort study described in detail elsewhere (see Kumari et al., 2011). This previous study identified changes both in symptoms and in neurobiology in the +CBTp group (n = 22) that were not found in the TAU group (n = 16). The present study focused on the +CBTp group and the TAU group was used for validation of the predictive model in an independent sample. Diagnosis was established at baseline using the Structured Clinical Interview for DSM-IV (SCID) (First et al., 1995). Symptoms were assessed using the Positive and Negative Syndrome Scale (PANSS) (Kay et al., 1987) for psychotic symptoms and the Beck Depression Inventory (BDI) (Beck et al., 1996) for depressive symptoms. All participants were scanned at the start of the study. Symptoms were reassessed after approximately six to eight months of TAU or TAU +CBTp. All participants were taking a stable dose of antipsychotic medication for a minimum of three months before the start of the study, which remained unchanged during the study. The study was approved by the joint research ethics committee of South London and Maudsley NHS trust and the Institute of Psychiatry in London (ref: 209/02). All participants provided written informed consent after explanation of the study procedures. Study procedures are reported in full elsewhere (Kumari et al., 2011).

### 2.2 Functional MRI task

A detailed description of the task and functional MRI acquisition can be found in the original study (Kumari et al., 2011). Participants performed an implicit facial affect task during the scanning session, in which they were presented with monochrome faces portraying fear (signalling sources of threat in the environment, i.e. indirect threat), anger (direct threat), happiness (prosocial affect), or neutral (ambiguous threat) expressions (Kumari et al., 2011). Participants had to press a button to indicate the gender of the face upon each facial presentation. The task was set up as a block design. Each block consisted of 8 trials in which the same facial expression was presented (3.75 s per face, 30 s in total). Participants were presented with four blocks of each condition (i.e. fearful, angry, happy, and neutral faces), fully counterbalanced. Between these blocks there were 4 baseline trials in which empty oval frames matched for luminance but without the face inside were shown (3.75 s per oval frame), and a left/right button press was required on each trial.

### 2.3 Functional MRI data acquisition and analysis

Data were collected on a widely available 1.5 Tesla General Electric Signa clinical system (echo time 40 ms, repetition time 3 s, flip angle 90°, field of view 240 mm, slice thickness 7.0 mm, interslice gap 0.7 mm). Two hundred and forty T2*-weighted images were acquired. Image pre-processing and data analyses were conducted using Statistical Parametric Mapping (SPM) version 12 (Wellcome Department of Imaging Neuroscience, www.fil.ion.ucl.ac.uk/spm). The multivariate pattern analysis was implemented in the Pattern Recognition for Neuroimaging Toolbox (PRoNTo) (www.mlnl.cs.ucl.ac.uk/pronto/) (Schrouff et al., 2013a), a machine learning toolbox that permits multivariate regression and classification analyses on neuroimaging data. Images were smoothed, normalized, slice time corrected and realigned. See the Supplementary Materials for further details on image pre-processing.

### 2.4 Activation associated with baseline symptoms

To optimize accuracy and generalisability of model predictions, feature reduction techniques were employed before training of the machine learning model (Mwangi et al., 2014). Feature selection involves selecting voxels that are considered informative and excluding those considered less or non-informative about predictions, resulting in less noise and increased predictive power. We constrained our analyses (Månsson et al., 2015; Yang et al., 2016) to symptom-locked activation, given our focus on predicting symptom improvement. We also report an unconstrained (whole-brain) analysis in the Supplementary Materials. In a first step, univariate regression analysis was used to identify clusters associated with baseline positive psychotic and depressive symptoms, with baseline symptom scores being regressed separately onto activation for each facial condition. The regions included in the functionally defined masks are presented in the Supplementary Tables 1 and 2. Functional masks were identified in the +CBTp group, which were then also independently evaluated in the separate TAU group, using a voxel-wise threshold of *p* < 0.001 with a cluster size of at least 10 active voxels (Takano et al., 2017). The resulting masks were used to constrain the multivariate pattern analysis that involved building of a predictive model at the group level to make predictions about symptom change (from pre- to post-intervention) at the individual patient level.

### 2.5 Predicting symptom improvement following CBTp

Multivariate regression models for neuroimaging data decode patterns of voxel values from the input images that continuously predict variability in the predicted variable (Schrouff et al., 2013a). Here, the inputs were contrast images for neutral, angry, fearful and happy faces to predict improvement in positive psychotic symptoms (PANSS-P) and depressive symptoms (BDI) for each patient. To assess the contribution of each condition to the prediction of improvement in positive psychotic and depressive symptoms, brain responses to the facial conditions were simultaneously assessed in a multiple kernel learning model (Schrouff et al., 2014). We also report follow-up analyses of models including only one facial condition in the Supplementary Materials. In the current study, the input voxels (features) were mean centred and normalized using the training data, and an indication of the model's generalizability was obtained using cross-validation and permutation testing. Cross-validation allows for assessment of the generalizability of the model using the available data by partitioning the data into training and testing sets (Mourao-Miranda et al., 2012). We report the Pearson's correlation coefficient (*r*), the Mean Squared Error (MSE), and the √MSE to assess the agreement between the predicted and actual symptom scores. The MSE reflects the sum of squared differences between the actual and predicted change in symptoms for each patient divided by the total number of patients (Schrouff et al., 2013a) and the √MSE can be interpreted as the standard deviation of the variance in symptom scores that is unexplained by the model (Willmott, 1981). A nested-k-fold scheme was used. Permutation testing over 1000 iterations was used to derive a *p*-value for the accuracy of the decision function (Mourao-Miranda et al., 2012). Further details on model optimization, the cross-validation scheme, and permutation testing are reported in the Supplementary Materials. To visualise the decision function, voxel-wise weights were computed for all significant models. Additionally, a list of regions ranked according to their contribution to the decision function was provided using the atlas (Schrouff et al., 2013b) as implemented within PRoNTo.

## 3 Results

### 3.1 Socio-demographic and clinical characteristics of the patient group

Demographic, clinical and task performance characteristics have been reported in full elsewhere (Kumari et al., 2011). In the +CBTp group, participants were on average 35.7 (SD = 7.8) years old and had completed an average of 13.9 (SD = 3.3) years of education. Twenty participants were on atypical and 2 were on both atypical and typical antipsychotics. In the TAU group, participants were on average 39.2 (SD = 9.37) years old and had completed an average of 13.6 (SD = 1.7) years of education. Fourteen participants were on atypical and 2 were on both atypical and typical antipsychotics. Both patient groups showed high gender discrimination accuracy (≥84.7%) across all conditions (i.e. fearful, angry, happy, and neutral faces) (Kumari et al., 2011). Performance was comparable to healthy controls, reported separately (Mason et al., 2016). Depressive symptoms were frequently experienced in addition to positive psychotic symptoms, with over 65% of participants experiencing symptoms in the mild to severe range. From pre-therapy to post-therapy time points, both groups showed variation in positive psychotic (+CBTp M = 3.2, SD = 3.9; TAU M = 0.5, SD = 4.0) and in depressive symptom scores (+CBTp M = 5.3, SD = 10.9, TAU M = 0.1, SD = 6.9). However, symptoms improved significantly in the +CBTp group only (Table 1).

**Table 1:** Means and standard deviations for positive psychotic and depressive symptoms pre- and post-therapy.

	**Depressive symptoms**^a^	**Positive psychotic symptoms**^b^
	Mean (SD)	Mean (SD)
**CBTp**		
Pre-therapy	16.2 (8.3)	18.1 (4.8)
Follow-up	11.5⁎ (9.9)	14.9⁎ (4.1)

**TAU**		
Pre-therapy	15.9 (10.4)	18.6 (3.2)
Follow-up	15.8 (12.1)	18.1 (3.3)

a Depressive symptoms were assessed using the Beck Depression Inventory (Beck et al., 1996).

b Positive psychotic symptoms were assessed using the positive psychotic symptom rating on the Positive and Negative Syndrome Scale (Kay et al., 1987).

⁎ Significant symptom reduction (*p* < 0.05) at follow up relative to baseline.

### 3.2 Predicting symptom improvement following CBTp

**Fig. 1:**
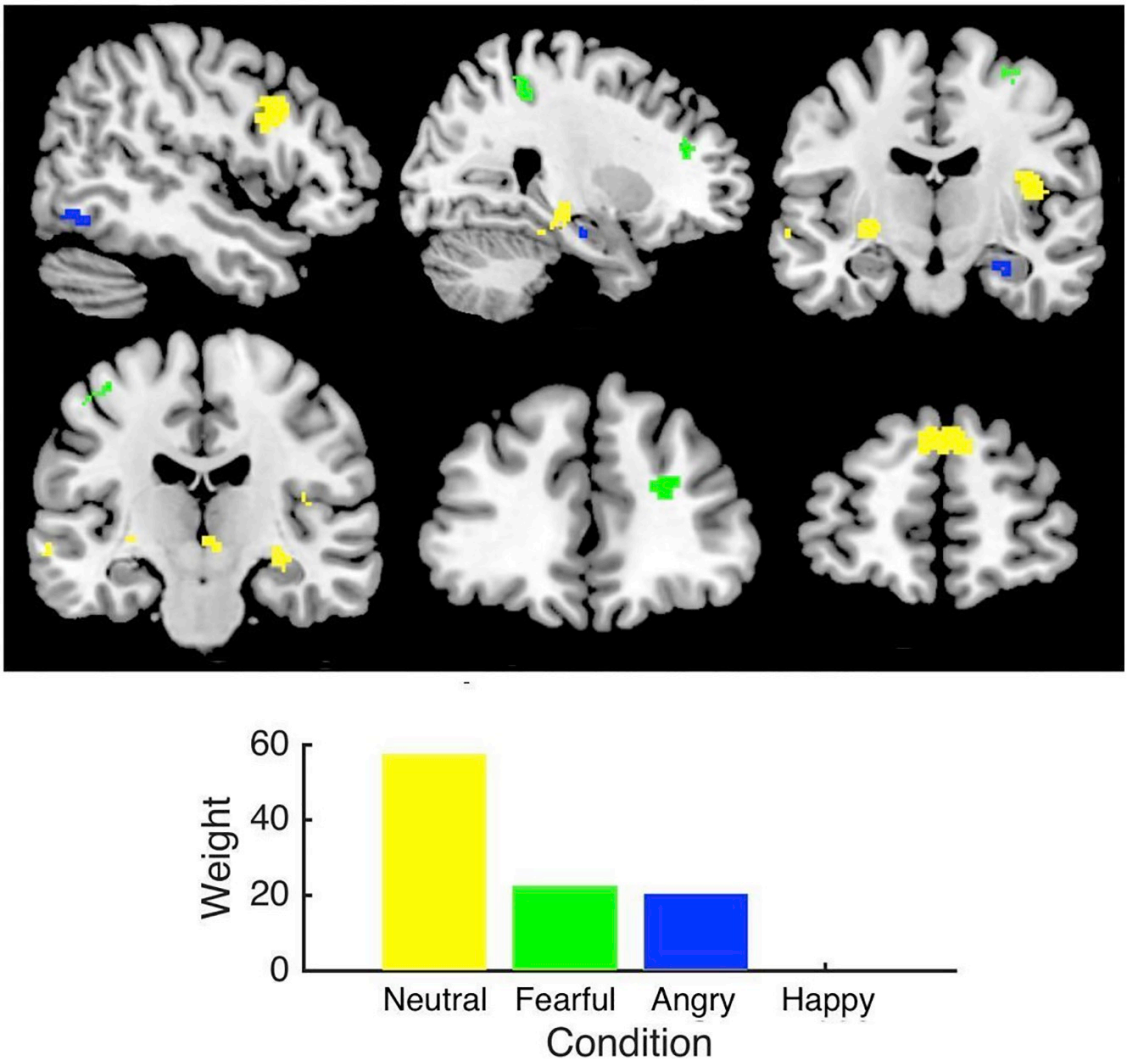
Baseline brain responses to threat-related affect (ambiguous ‘neutral’ faces that are typically perceived as threatening in psychosis, in addition to fearful and angry faces) predict improvement in positive psychotic symptoms following CBTp. The multiple kernel learning model is significant (*r* = 0.63, *p* = 0.003). The bar graph shows the relative contribution of each facial condition to the decision function. Top, from left to right: x = 48, 26, y = −12. Bottom, from left to right: y = −18, 39, 56.

Improvement in positive psychotic symptoms was uniquely predicted by activation elicited by all types of threat-related affect (i.e. ambiguous ‘neutral’ faces that are often perceived as threatening in psychosis, in addition to angry, and fearful faces) (*r* = 0.63, *p* = 0.003) (Table 2). Activation in frontal, sensorimotor, and hippocampal regions contributed most strongly to the predictive model (Table 3). However, the profile of activation was confined to fewer regions for angry and fearful faces compared to the more widespread profile of activation for neutral faces (Supplementary Table 3). Activation elicited by neutral faces contributed most strongly to the model followed by equivalent contributions from activation for angry and fearful faces (Fig. 1). Follow-up analyses of models including only one facial condition revealed that activation elicited by angry, fearful, and neutral faces was predictive of improvement in positive psychotic symptoms, but activation elicited by happy faces was not (Supplementary Table 4).

**Table 2:** Predictive accuracy of multivariate models for response to cognitive behavioural therapy for psychosis.

	*r*	*P*_(*r*)_	MSE	*P*_(MSE)_	√MSE
**Positive psychotic symptoms**^a^	**0.63**	**0.003**⁎	**8.65**	**0.003**⁎	**2.94**
Neutral faces (57.6%)					
Angry faces (22.2%)					
Fearful faces (20.2%)					
Happy faces (0%)					

**Depressive symptoms**^b^	**0.31**	**0.05**⁎	**103.73**	**0.04**⁎	**10.18**
Fearful faces (73.1%)					
Happy faces (25.7%)					
Neutral faces (1.2%)					
Angry facest^c^					

Neutral faces (ambiguous threat). Angry faces (direct threat). Fearful faces (indirect threat). Happy faces (prosocial affect). Abbreviations: MSE, mean squared error.

a Positive psychotic symptoms were assessed using the positive psychotic symptom rating on the Positive and Negative Syndrome Scale (Kay et al., 1987).

b Depressive symptoms were assessed using the Beck Depression Inventory (Beck et al., 1996).

c Not included in multivariate analysis because no symptom-locked activity at baseline.

⁎ *p* ≤ 0.05.

**Table 3:** Top 3 predictors for positive psychotic and depressive symptoms and their relative weights in predictive power (percentage of the total weights in the decision function); clusters <10 active voxels excluded.

Anatomical region	MNI coordinates			Brodmann area	weight (%)	size (voxels)
	x	y	z			
**Positive psychotic symptoms**						
*‘Neutral’ faces (57*.*6%)*						
R superior frontal gyrus	14	25	51	8	5.4	21
R cerebellum	10	−76	−22		5.4	46
L supplementary motor area	−42	−12	16	6	4.4	26
*Fearful faces (22*.*2%)*						
L precentral gyrus	−26	32	26	4	9.9	81
L superior frontal gyrus	−32	−10	58	6	9.9	40
R middle cingulum	10	22	36	8	7.8	16
*Angry faces (20*.*2%)*						
R inferior occipital gyrus	42	−76	−12	19	44.6	39
R hippocampus	28	−12	−20		38.5	23
R inferior temporal gyrus	48	−64	−10	37	16.9	21
*Happy faces (0%)*						
R inferior frontal gyrus	56	32	8	45	0	14

**Depressive symptoms**^a^						
*Fearful faces (73*.*1%)*						
L superior frontal gyrus	−10	28	52	8	74.0	20
*Happy faces (25*.*7%)*						
L inferior frontal gyrus	−52	24	28	44	12.7	84
R precentral gyrus	42	0	52	6	9.8	21
L fusiform gyrus	−40	−38	−20	37	5.8	62
*‘Neutral’ faces (1*.*2%)*						
R hippocampus	28	−22	−14	54	13.7	25
L precentral gyrus	−10	−34	−30	6	9.6	48
R parahippocampal gyrus	28	−11	−27	36	7.1	16

Neutral faces (ambiguous threat). Angry faces (direct threat). Fearful faces (indirect threat). Happy faces (prosocial affect).

a No predictors for angry faces because no symptom-locked activity at baseline.

**Fig. 2:**
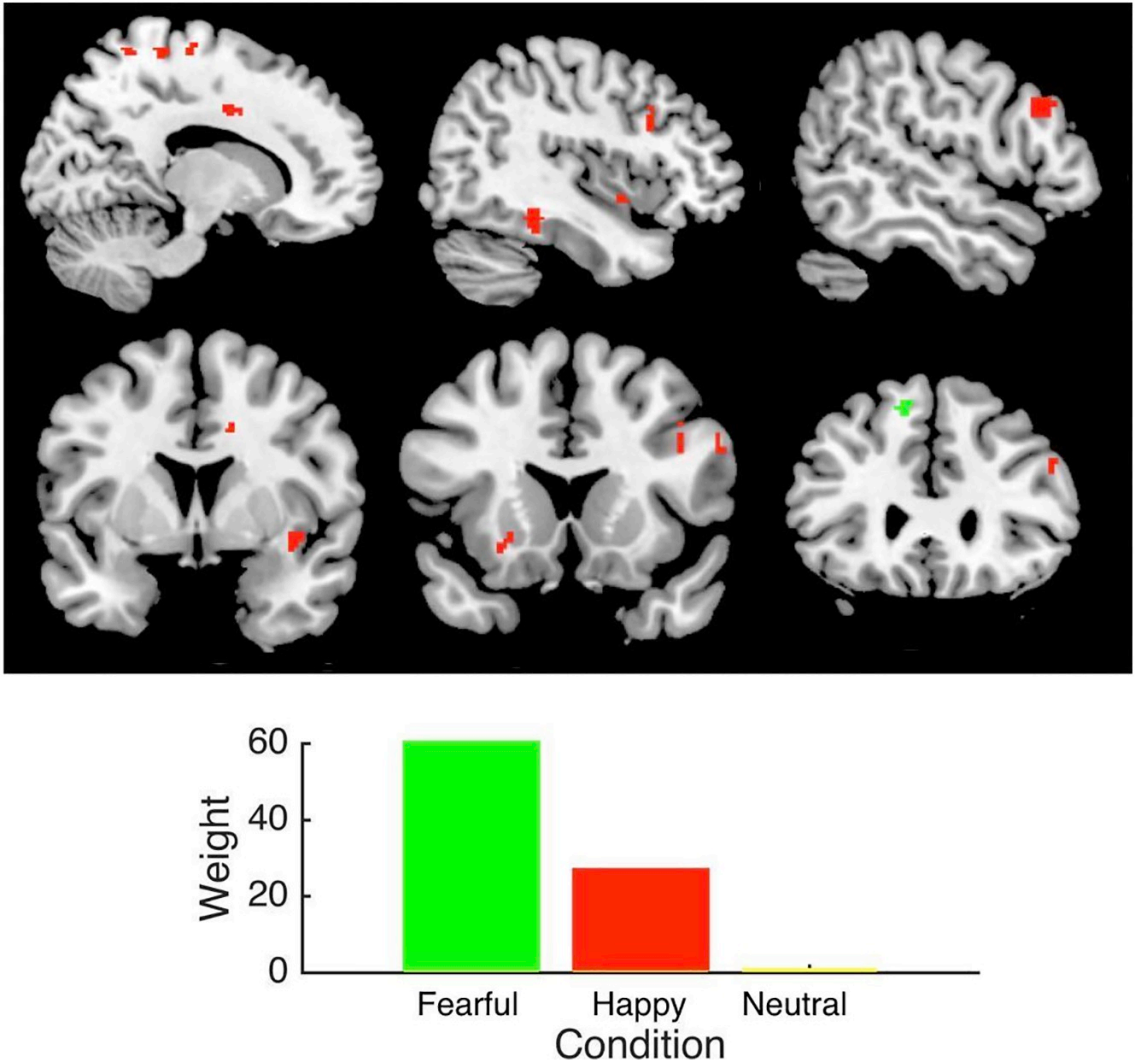
Baseline brain responses to fearful faces (indirect threat) and happy faces (prosocial affect) predict improvement in depressive symptoms following CBTp. The multiple kernel learning model is significant (*r* = 0.31, *p* = 0.05). The bar graph shows the relative contribution of each condition to the decision function. Top, from left to right: x = 14, 41, 53. Bottom, from left to right: y = 4, 17, 29.

Improvement in depressive symptoms was uniquely predicted by activation elicited by fearful and happy faces (*r* = 0.31, *p* = 0.05) (Table 2). Activation in frontal and motor regions contributed most strongly to the predictive model (Table 3). Activation in the superior frontal gyrus solely contributed to the predictive model for fearful faces whereas a widespread pattern of activation across frontal, sensorimotor, limbic, and occipital regions contributed to the predictive model for happy faces (Supplementary Table 5). Activation elicited by fearful faces contributed most strongly to the model followed by activation for happy faces (Fig. 2). Follow-up analyses of models including only one facial condition revealed that activation elicited by fearful and happy faces was predictive but activation elicited by neutral faces was not (Supplementary Table 4). Activation elicited by angry faces could not be assessed since no activation was associated with symptoms at baseline.

### 3.3 Assessment of predictive models in independent treatment-as-usual group

The assessment of the predictive models in the TAU group revealed that changes in both positive psychotic (*r* = −0.39, *p* = 0.46) and depressive symptoms (*r* = −0.05, *p* = 0.31) following TAU could not be predicted using the multiple kernel learning model (Table 4). Whilst the pattern was similar in terms of which facial conditions predicted changes in positive psychotic and depressive symptoms, with activation for neutral and fearful faces being the strongest predictor for changes in positive psychotic and depressive symptoms, respectively, the overall model did not reach significance (Supplementary Figs. 1, 2).

**Table 4:** Assessment of predictive models in independent treatment-as-usual group.

	*r*	*P*_(*r*)_	MSE	*P*_(MSE)_	√MSE
**Positive psychotic symptoms**^a^	**−0.39**	**0.46**	**21.21**	**0.67**	**4.60**
Neutral faces (62.9%)					
Fearful faces (33.8%)					
Happy faces (3.3%)					
Angry faces (0%)					

**Depressive symptoms**^b^	**−0.05**	**0.31**	**52.24**	**0.67**	**7.23**
Fearful faces (61.1%)					
Happy faces (26.5%)					
Neutral faces (11.9%)					
Angry faces^c^					

Neutral faces (ambiguous threat). Angry faces (direct threat). Fearful faces (indirect threat). Happy faces (prosocial affect). Abbreviations: MSE, mean squared error.

a Positive psychotic symptoms were assessed using the positive psychotic symptom rating on the Positive and Negative Syndrome Scale (Kay et al., 1987).

b Depressive symptoms were assessed using the Beck Depression Inventory (Beck et al., 1996).

c Not included in multivariate analysis because no symptom-locked activity at baseline.

## 4 Discussion

This proof-of-concept study set out to establish novel predictors from neural processing of social affective information by applying multivariate pattern analysis to pre-treatment functional MRI data. This research strategy bridges the gap between studies revealing associations between brain changes and CBTp outcomes (Kumari and Terca, 2017) and potentially clinically useful biomarkers that can inform understanding of treatment mechanisms and provide a step towards predictions of outcome and treatment planning for individual patients. The findings showed, to our knowledge for the first time, that machine learning methods can be used to build a model that can predict response to CBTp for each patient from pre-therapy neural responses to social affective information. In line with our hypotheses, we found a double dissociation between the valence of social affective information and the type of symptoms predicted. Whereas the brain activation pattern in response to angry (direct threat) and neutral (ambiguous threat) faces uniquely predicted improvement in positive psychotic symptoms, the brain activation pattern in response to happy faces (prosocial affect) uniquely predicted improvement in depressive symptoms. In contrast, neural responses to fearful faces (indirect threat) predicted improvement in both positive psychotic and depressive symptoms.

These findings also highlight the potential for this approach to inform clinical decision-making. Caveated by a need for more research with larger samples, it may be possible to use limited predicted symptom improvement for a given patient to better tailor their treatment plan. One possibility is that CBTp may be augmented with other interventions (Drake et al., 2014). Alternatively, it may be possible to use baseline neurobiological responses to social affect to plan and tailor the focus of therapy, such as spending more time engaging the client or a specific focus on modifying bias towards threatening social information. Considering the protracted duration of psychological therapy in which clinical signs of improvement may not be apparent for many weeks from the start of therapy, a robust biomarker may also act as an incentive to motivate the patient and increase compliance in addition to increasing the response rate in a stratified population. The clinical promise of this approach is underlined by the finding that clinically relevant stimuli can be used as assays for making specific predictions about different symptom domains and is supported by the growing interest in machine learning to optimize treatment outcomes (Månsson et al., 2015; Redlich et al., 2016; van Waarde et al., 2015).

It is interesting that processing of ambiguous ‘neutral’ faces was a stronger predictor of treatment response for positive psychotic symptoms than more directly threatening angry faces. One possible explanation for this finding is that processing of ambiguous stimuli particularly involves activation related to appraisals that are re-framed as part of CBTp. Namely, ambiguous stimuli have sensory characteristics that are less defined, thereby allowing appraisals to have a stronger influence on their perception and interpretation. Evidence suggests that patients with psychosis appraise even mildly anomalous experiences as more threatening compared to healthy individuals, remitted patients, and individuals with similar psychotic experiences but without a need for care (Ward et al., 2014; Underwood et al., 2016b; Peters et al., 2017). Therefore, processing of ambiguous stimuli might most strongly reflect threatening appraisals. Neural evidence for an over- or misattribution of threat involves elevated activation to neutral or ambiguous stimuli (Potvin et al., 2016; Underwood et al., 2015; Lakis and Mendrek, 2013), which has been attributed to aberrant salience perception (Kapur, 2003). This suggests that neutral or ambiguous stimuli may convey subtle information to which patients with psychosis are particularly sensitive, resulting in brain responses to ambiguous facial expressions not only reliably differentiating patients from healthy controls (Potvin et al., 2016) but also those who will respond well from those who will respond poorly. Processing of ambiguous stimuli might therefore most strongly reflect threatening appraisals. Since recent studies have shown that appraisals of symptoms mediate changes in outcome (Birchwood et al., 2017) the finding that threat-related activation, in particular, activation in response to ambiguous ‘neutral faces’, predicted improvement in positive psychotic symptoms suggests that CBTp most likely improves psychotic symptoms in those endorsing more threatening appraisals of neutral or ambiguous stimuli (Underwood et al., 2015). However, this claim is at present speculative and requires more sophisticated measures to be substantiated.

Brain activation in response to prosocial affect (happy faces) was unique in predicting improvement in depressive symptoms and not positive psychotic symptoms. However, brain activation in response to fearful faces emerged as an additional, and stronger, predictor of improvement in depressive symptoms than brain activation in response to happy faces. Enhanced brain responses to fearful faces were found to be associated with baseline depressive symptoms in this clinical group (Kumari et al., 2016) and reduced following CBTp in previous analyses (Kumari et al., 2011). Additionally, the finding that processing of both happy and fearful faces could predict improvement in depressive symptoms is in line with neuroimaging studies revealing both hypo-responses to positive and hyper-responses to negative facial expressions in depression (Stuhrmann et al., 2011). These findings suggest that CBTp most likely improves depressive symptoms in those with mood-congruent processing biases that are reduced by exploring the impact of thoughts and behaviours on depressive symptoms.

Supporting our predictions, activity in the dorsolateral prefrontal cortex was important for predicting improvement in both positive psychotic and depressive symptoms. However, contrary to our hypothesis, the hippocampus emerged as a stronger predictor of improvement in positive psychotic symptoms than the amygdala. The dorsolateral prefrontal cortex and hippocampus are part of the dorsal system, which is involved in the regulation of emotions (Underwood et al., 2016a). The predictive power of activation in the dorsal system supports the idea that CBTp may improve positive psychotic symptoms by facilitating patients' ability to re-appraise their threatening experiences (Underwood et al., 2016a). Interestingly, a number of sensorimotor regions, including the supplementary motor area, as well as visual regions, including the occipital and fusiform gyrus, also emerged as important predictors of improvement in both positive psychotic and depressive symptoms. Sensorimotor regions are, together with frontal regions, important for generating situation-specific behavioural responses (Janak and Tye, 2015). The contribution of sensorimotor regions to predictions suggests an important role for behavioural techniques in improving both positive psychotic and depressive symptoms, by promoting change in unhelpful behaviours that contribute to the maintenance of delusional beliefs and mood worsening. Together, these findings suggest that a combination of threat-regulation and action preparation, as well as higher-order cognitive processes are key to predicting improvement in positive psychotic and depressive symptoms.

Whilst the dorsolateral PFC was an important predictor of symptom improvement, it was not a stronger predictor than a large number of regions across sensorimotor and midbrain regions. One possibility is that the present task did not actively recruit higher-order cognitive processes such as reappraisal because participants were not explicitly prompted to process the affective component of the stimuli. Additionally, these findings need to be caveated by the focus on symptom-locked activation, which omitted other areas from the analysis. A Supplementary whole-brain analysis addressed this issue, and although the pattern was similar in terms of which facial conditions predicted changes in symptoms, the overall model did not reach significance (Supplementary Tables 6, 7). Despite these constraints, the finding that activation in the dorsal system, including the dorsolateral PFC and hippocampus, was important for predicting improvement in positive psychotic symptoms, supports the idea that CBTp facilitates reappraisal through resources in higher-order brain regions that regulate those involved in threat and salience detection (Mason et al., 2016).

Although the present study successfully predicted symptom improvement, future studies including additional measurements would provide more encompassing predictions of treatment effects. The PANSS as an instrument has been criticised for only providing a measure of the presence and severity of psychotic symptoms, rather than the considerable variation in impact and quality of these experiences across individuals (Birchwood and Trower, 2012). This is in line with previous work, which has shown that CBTp-led changes in brain responses to indirect threat were uncorrelated with the PANSS (Kumari et al., 2011). Future studies should explore measures that consider symptom dimensions such as power beliefs and distress to provide further insight into the predictors of treatment outcomes. Additionally, there is promise in exploring whether threat processing as a predictor is a state or trait marker. State and trait features are likely to influence the development and maintenance of threatening appraisals along different pathways, including attentional, attributional, and reasoning biases as well as safety behaviours (Underwood et al., 2016a). Elucidating these features might help guide effective treatment strategies at the individual patient level.

Whilst the model identified predicted response to CBTp, it did not predict response in the independent TAU group. Caveated by the limited change in symptoms in the TAU group, these results may speak to the specificity of the findings in predicting response to CBTp rather than symptom changes per se. Future research with two active treatment groups can provide further insight into the specific predictors of response to CBTp. Additionally, the specificity of the facial conditions for predicting improvement in different symptom domains is further underlined by the similar contribution of the facial conditions to predictions in the +CBTp and independent TAU group (Supplementary Figs. 1, 2).

Although the sample size of the present study is comparable to other machine learning studies in psychiatry (Månsson et al., 2015; Yang et al., 2016; Johnston et al., 2015), future studies with larger samples are warranted. The patient group reported here is likely to be representative of routine clinical practice, having been recruited from a clinic as part of routine care. However, further research with larger and independent samples of patients receiving CBTp should be undertaken to further establish the utility of machine learning approaches to predicting treatment response. Additionally, the use of higher resolution functional MRI to investigate subcortical predictors should be further explored as well as the inclusion of behavioural and clinical measures into predictive models considering that the most optimal predictions likely require different sources of information (McMahon, 2014).

In summary, the present study supports the utility of machine learning methods to predict how people will respond when offered CBTp. The clinical utility of this approach is further underscored by the finding that neural responses to threat-related affect (i.e. ambiguous ‘neutral’ faces that are typically perceived as threatening in psychosis, in addition to angry and fearful faces) specifically predicted improvement in positive psychotic symptoms, whereas neural responses to fearful and happy faces predicted improvement in depressive symptoms. These findings suggest that CBTp most likely improves psychotic and affective symptoms in those endorsing more threatening appraisals and mood-congruent processing biases, respectively, which are explored and reframed as part of the therapy. Caveated by further research in larger and independent samples of patients receiving CBTp, baseline activation patterns in response to social affective information may assist in individual therapy formulations by informing the focus of the therapy on threat or mood-congruent processing biases that are addressed through different techniques including the generation of alternative explanations for psychotic experiences and the exploration of mood on thinking styles. Machine learning methods may therefore become a valuable tool for mapping the neural correlates of these biases to make predictions about treatment outcomes for each patient. This promising approach may be further refined, for example by including additional predictors, such as structural and connectivity measures. It is our hope that these methods may ultimately go beyond treatment selection and be used to tailor and refine the psychological intervention offered to individual patients.

## Conflict of interest

None of the authors report any financial interests or potential conflict of interest.

## Funding

This work was supported by the Wellcome Trust (Senior Research Fellowship in Basic Biomedical Science to V.K., grant no. 067427).

## Appendix A Supplementary data

Supplementary material Image 1
