# Supplementary material for: Using fMRI and machine learning to predict symptom improvement following cognitive behavioural therapy for psychosis
Source: Neuroimage Clin. 2018 Oct 10;20:1053–61. doi: 10.1016/j.nicl.2018.10.011 (PMC6197386; doi:10.1016/j.nicl.2018.10.011)
Supplement: Supplementary file 1 — Supplementary material [file mmc1.doc]

**SUPPLEMENTARY MATERIAL**

**Materials & methods**

**Image pre-processing**

Images were pre-processed by taking the 240 volume functional time series of each participant and applying motion correction, transformation into stereotactic space (Montreal Neurological Institute, MNI), and smoothing with an 8-mm full width at half maximum Gaussian filter band pass.

**Model optimization**

A Multiple Kernel Learning model (Schrouff et al., 2014) was selected to simultaneously assess the contribution of each affective condition to the prediction. A Kernel Ridge Regression model (Shawe-Taylor et al., 2004) was selected to optimise predictions for each separate affective condition since kernel methods have an effective way of dealing with high-dimensional data (Chu et al., 2010; Schrouff et al., 2013). The hyper-parameters were optimised using a 0.1 1 100 range. For better performance of the model, a first-level mask was created that excludes voxels with NaN (Not a Number) values from the analysis. To create this mask, a template mask in PRoNTo (SPM_mask_noeyes) was updated with the beta images of the participants.

**Functionally defined masks using activation associated with baseline symptoms**

A widespread pattern of activation elicited by ambiguous ‘neutral’ faces that are perceived as threatening in psychosis across frontal, limbic, and sensorimotor areas was positively associated with baseline positive psychotic symptoms (Supplementary Table 1). Activation elicited by angry faces in the right inferior temporal gyrus, occipital gyrus, and hippocampus was also positively associated with baseline positive psychotic symptoms. Activation elicited by fearful faces across sensorimotor and frontal regions was negatively associated with baseline positive psychotic symptoms. Activation elicited by happy faces in the right inferior frontal gyrus was positively associated with baseline positive psychotic symptoms.

Supplementary Table 1. Areas activating in response to facial affect that covary with current positive psychotic symptoms. Voxel threshold *p* < .001; clusters < 10 active voxels excluded.

|  |  |  | | | | |  |  |  | |
| --- | --- | --- | --- | --- | --- | --- | --- | --- | --- | --- |
| Anatomical region |  | MNI coordinates | | | | |  | Cluster size | Z | |
|  |  | x |  | y |  | z |  |  |  |  |
|  |  |  |  |  |  |  |  |  |  |  |
| **Neutral faces** |  |  |  |  |  |  |  |  |  |  |
| *Positive association* |  |  |  |  |  |  |  |  |  |  |
| L/R olfactory gyrus extending into caudate |  | -4 |  | 14 |  | 10 |  | 467 |  | 4.39 |
| L/R hippocampus extending into temporal gyrus |  | 4 |  | -28 |  | -2 |  | 668 |  | 4.26 |
| R temporal gyrus extending into insula |  | 42 |  | -78 |  | -2 |  | 226 |  | 4.03 |
| L cerebellum extending into vermis |  | -4 |  | -60 |  | -26 |  | 234 |  | 3.87 |
| L/R inferior, superior frontal gyrus |  | 48 |  | 12 |  | 30 |  | 339 |  | 3.81 |
| L pallidum extending into temporal gyrus |  | -28 |  | -14 |  | -2 |  | 66 |  | 3.61 |
| L insula extending into precentral gyrus |  | -36 |  | -4 |  | 14 |  | 23 |  | 3.50 |
| L/R precentral gyrus |  | 52 |  | 2 |  | 44 |  | 28 |  | 3.43 |
| L/R cerebellum extending into fusiform gyrus |  | 18 |  | -34 |  | -26 |  | 47 |  | 3.41 |
| L/R superior frontal gyrus |  | -14 |  | 16 |  | 52 |  | 37 |  | 3.29 |
|  |  |  |  |  |  |  |  |  |  |  |
| **Angry faces** |  |  |  |  |  |  |  |  |  |  |
| *Positive association* |  |  |  |  |  |  |  |  |  |  |
| R inferior temporal, inferior occipital gyrus |  | 50 |  | -66 |  | -12 |  | 39 |  | 4.05 |
| R inferior occipital gyrus |  | 42 |  | -80 |  | -12 |  | 21 |  | 3.76 |
| R hippocampus |  | 30 |  | -12 |  | -20 |  | 23 |  | 3.55 |
|  |  |  |  |  |  |  |  |  |  |  |
| **Fearful faces** |  |  |  |  |  |  |  |  |  |  |
| *Negative association* |  |  |  |  |  |  |  |  |  |  |
| R middle frontal gyrus extending into precentral gyrus |  | 30 |  | -12 |  | 62 |  | 35 |  | 4.28 |
| R superior, inferior parietal gyrus extending into supramarginal gyrus |  | 28 |  | -38 |  | 50 |  | 77 |  | 4.18 |
| L postcentral, precentral gyrus |  | -40 |  | -18 |  | 58 |  | 37 |  | 3.91 |
| R superior, middle frontal gyrus |  | 26 |  | 38 |  | 20 |  | 57 |  | 3.87 |
| L superior frontal gyrus extending into cingulum |  | -12 |  | 22 |  | 34 |  | 31 |  | 3.65 |
| R supplementary motor area extending into superior frontal gyrus |  | 18 |  | 20 |  | 52 |  | 54 |  | 3.48 |
|  |  |  |  |  |  |  |  |  |  |  |
| **Happy faces**  *Positive association* |  |  |  |  |  |  |  |  |  |  |
| R inferior frontal gyrus |  | 56 |  | 32 |  | 8 |  | 14 |  | 3.45 |
|  |  |  |  |  |  |  |  |  |  |  |

Activation elicited by ambiguous ‘neutral’ faces across limbic and sensorimotor areas was positively associated with baseline depressive symptoms (Supplementary Table 2). Activation elicited by angry faces was not associated with baseline depressive symptoms, whereas activation elicited by fearful faces in the left superior frontal gyrus was positively associated with baseline depressive symptoms. Activation elicited by happy faces across frontal, sensorimotor, and midbrain regions was negatively associated with baseline depressive symptoms.

Supplementary Table 2. Areas activating in response to facial affect that covary with current depressive symptoms. Voxel threshold *p* < .001; clusters < 10 active voxels excluded.a

|  |  |  | | | | |  |  |  | |
| --- | --- | --- | --- | --- | --- | --- | --- | --- | --- | --- |
| Anatomical region |  | MNI coordinates | | | | |  | Cluster size | Z | |
|  |  | x |  | y |  | z |  |  |  |  |
|  |  |  |  |  |  |  |  |  |  |  |
| **Neutral faces** |  |  |  |  |  |  |  |  |  |  |
| *Positive association* |  |  |  |  |  |  |  |  |  |  |
| L parahippocampal gyrus extending into hippocampus |  | -30 |  | -12 |  | -24 |  | 44 |  | 3.92 |
| L pallidum, putamen |  | -14 |  | 4 |  | -4 |  | 11 |  | 3.73 |
| L amygdala |  | -30 |  | 0 |  | -24 |  | 13 |  | 3.37 |
| L cerebellum extending into fusiform gyrus |  | -36 |  | -48 |  | -28 |  | 24 |  | 3.36 |
|  |  |  |  |  |  |  |  |  |  |  |
| **Fearful faces** |  |  |  |  |  |  |  |  |  |  |
| *Positive association* |  |  |  |  |  |  |  |  |  |  |
| L superior frontal gyrus |  | -12 |  | 30 |  | 52 |  | 21 |  | 3.59 |
|  |  |  |  |  |  |  |  |  |  |  |
| **Happy faces** |  |  |  |  |  |  |  |  |  |  |
| *Negative association* |  |  |  |  |  |  |  |  |  |  |
| L paracentral lobule extending into postcentral gyrus |  | -16 |  | -42 |  | 64 |  | 225 |  | 4.51 |
| R middle, inferior frontal gyrus |  | 52 |  | 24 |  | 26 |  | 80 |  | 4.15 |
| R paracentral lobule extending into precentral gyrus |  | 12 |  | -20 |  | 68 |  | 40 |  | 3.96 |
| R inferior temporal, fusiform gyrus |  | 40 |  | -38 |  | -18 |  | 74 |  | 3.86 |
| L supplementary motor area, precentral gyrus |  | -14 |  | -4 |  | 68 |  | 16 |  | 3.76 |
| R paracentral lobule extending into precentral gyrus |  | 14 |  | -34 |  | 68 |  | 20 |  | 3.66 |
| R middle cingulum |  | 12 |  | 0 |  | 36 |  | 24 |  | 3.65 |
| R insula |  | 40 |  | 4 |  | -8 |  | 28 |  | 3.64 |
| L lingual gyrus extending into superior temporal gyrus |  | -22 |  | -56 |  | -4 |  | 33 |  | 3.58 |
| L middle frontal, precentral gyrus |  | -42 |  | 0 |  | 52 |  | 20 |  | 3.45 |
| R precuneus extending into postcentral gyrus |  | 12 |  | -52 |  | 66 |  | 20 |  | 3.42 |
| L putamen |  | -24 |  | 16 |  | -10 |  | 13 |  | 3.38 |
| R middle frontal gyrus extending into postcentral gyrus |  | 42 |  | 18 |  | 32 |  | 36 |  | 3.29 |
| L fusiform, inferior temporal gyrus |  | -42 |  | -46 |  | -8 |  | 11 |  | 3.25 |
|  |  |  |  |  |  |  |  |  |  |  |

^a^No clusters for direct threat because no symptom-locked activity at baseline.

**Cross validation & permutation testing**

5-fold was selected for the inner loop and 10-fold for the outer loop (Svetnik et al., 2004). To assess the significance, the multivariate pattern analysis model was rerun 1000 times. The number of times the permuted value was greater than, (or regarding the mean squared error between true and predicted clinical scores, less than), or equal to, the true value, was divided by 1000 to get an estimated *p*-value for the Pearson’s correlation coefficient (*r*) and the mean squared error.

**Results**

**Predicting symptom improvement following CBTp**

Improvement in positive psychotic symptoms was best predicted by activation elicited by threat-related affect (ambiguous ‘neutral’ faces in addition to angry and fearful faces). A widespread pattern of activation for ambiguous ‘neutral’ faces across frontal, sensorimotor and limbic regions as well as more focal patterns of activation for angry and fearful faces contributed to the predictive model for positive psychotic symptoms (Supplementary Table 3). Separate assessment of each affective condition revealed that improvement in positive psychotic symptoms could be predicted by activation elicited by ambiguous ‘neutral’ faces (*r*=0.61, *p*=0.002), angry faces (*r*=0.54, *p*=0.01) and fearful faces (*r*=0.32, *p*=0.03), but not by happy faces (Supplementary Table 4).

Supplementary Table 3. Top 20 predictors of improvement in positive psychotic symptoms and their relative weights in predictive power (percentage of the total weights in the decision function); clusters < 10 active voxels excluded.

|  |  |  |  | | | | | |  |  | | | |  | | |
| --- | --- | --- | --- | --- | --- | --- | --- | --- | --- | --- | --- | --- | --- | --- | --- | --- |
| Anatomical region | | | | MNI coordinates | | |  | Brodmann area | | | | weight (%) | | | size (voxels) | |
|  | | | | x | y | z |  | | | |  | |  | | |  |
|  | | | |  |  |  |  | | | |  | |  | | |  |
| **Neutral faces (57.6%)** | | | |  |  |  |  | | | |  | |  | | |  |
| R superior frontal gyrus | | | | 14 | 25 | 51 | 8 | | | |  | | 5.4 | | | 21 |
| R cerebellum | | | | 10 | -76 | -22 |  | | | |  | | 5.4 | | | 46 |
| L supplementary motor area | | | | -42 | -12 | 16 | 6 | | | |  | | 4.4 | | | 26 |
| L cerebellum | | | | -44 | -70 | -8 |  | | | |  | | 4.3 | | | 35 |
| L insula | | | | -38 | -12 | 14 | 13 | | | |  | | 4.2 | | | 85 |
| L inferior frontal gyrus | | | | -46 | 12 | 28 | 44 | | | |  | | 4.0 | | | 114 |
| R inferior frontal gyrus | | | | 46 | 10 | 28 | 44 | | | |  | | 3.8 | | | 28 |
| R precentral gyrus | | | | 46 | -9 | 46 | 6 | | | |  | | 3.3 | | | 12 |
| R fusiform gyrus | | | | 34 | -36 | -20 | 37 | | | |  | | 3.1 | | | 203 |
| R middle temporal gyrus | | | | 62 | -16 | -3 | 22 | | | |  | | 2.1 | | | 16 |
| L amygdala | | | | -28 | -5 | -15 |  | | | |  | | 1.9 | | | 28 |
| L parahippocampal gyrus | | | | -26 | -22 | -19 | 36 | | | |  | | 1.5 | | | 94 |
| R insula | | | | 38 | 4 | 15 | 13 | | | |  | | 1.7 | | | 20 |
| cerebellar vermis | | | | 0 | -60 | -28 |  | | | |  | | 1.4 | | | 49 |
| L fusiform | | | | -40 | -62 | -8 | 37 | | | |  | | 1.3 | | | 67 |
| L precentral gyrus | | | | -46 | -4 | 46 | 6 | | | |  | | 1.3 | | | 665 |
| R caudate | | | | 10 | 18 | 4 |  | | | |  | | 1.2 | | | 170 |
| R hippocampus | | | | 36 | -32 | -8 |  | | | |  | | 1.1 | | | 14 |
| L middle frontal gyrus | | | | -6 | 54 | 32 | 9 | | | |  | | 1.0 | | | 71 |
| L thalamus | | | | 8 | -26 | 0 |  | | | |  | | 0.9 | | | 14 |
|  | | | |  |  |  |  | | | |  | |  | | |  |
| **Fearful faces (22.2%)** | | | |  |  |  |  | | | |  | |  | | |  |
| L precentral gyrus | | | | -26 | 32 | 26 | 4 | | | |  | | 9.9 | | | 81 |
| L superior frontal gyrus | | | | -32 | -10 | 58 | 6 | | | |  | | 9.9 | | | 40 |
| R middle cingulum | | | | 10 | 22 | 36 | 8 | | | |  | | 7.8 | | | 16 |
| L middle frontal gyrus | | | | -30 | 34 | 24 | 10 | | | |  | | 5.1 | | | 16 |
| L parietal gyrus | | | | -26 | -40 | 52 | 5 | | | |  | | 4.6 | | | 36 |
| R precentral gyrus | | | | 38 | -18 | 58 | 4 | | | |  | | 4.6 | | | 17 |
| R postcentral gyrus | | | | -44 | -22 | 56 | 1 | | | |  | | 4.2 | | | 20 |
| L supramarginal gyrus | | | | -30 | -42 | 52 | 7 | | | |  | | 1.4 | | | 11 |
|  | | | |  |  |  |  | | | |  | |  | | |  |
| **Angry faces (20.2%)** | | | |  |  |  |  | | | |  | |  | | |  |
| R inferior occipital gyrus | | | | 42 | -76 | -12 | 19 | | | |  | | 44.6 | | | 39 |
| R hippocampus | | | | 28 | -12 | -20 |  | | | |  | | 38.5 | | | 23 |
| R inferior temporal gyrus | | | | 48 | -64 | -10 | 37 | | | |  | | 16.9 | | | 21 |
|  | | | |  |  |  |  | | | |  | |  | | |  |

Supplementary Table 4. Predictive accuracy of each affective condition for response to cognitive behavioural therapy for psychosis.

|  | *r* |  | *P*(*_r_*) |  | MSE |  | *P*_(MSE)_ |
| --- | --- | --- | --- | --- | --- | --- | --- |
|  |  |  |  |  |  |  |  |
| **Positive psychotic symptoms**^a^ |  |  |  |  |  |  |  |
| Neutral faces | 0.61 |  | 0.002* |  | 9.01 |  | 0.002* |
| Angry faces | 0.54 |  | 0.01* |  | 10.40 |  | 0.01* |
| Fearful faces | 0.32 |  | 0.03* |  | 14.44 |  | 0.07 |
| Happy faces | -0.27 |  | 0.44 |  | 17.82 |  | 0.79 |
|  |  |  |  |  |  |  |  |
| **Depressive symptoms**^b^ |  |  |  |  |  |  |  |
| Fearful faces | 0.59 |  | 0.002* |  | 75.05 |  | 0.003* |
| Happy faces | 0.32 |  | 0.05* |  | 102.93 |  | 0.04* |
| Ambiguous ‘neutral faces’ | -0.15 |  | 0.29 |  | 122.47 |  | 0.13 |
| Angry faces^c^ | - |  | - |  | - |  | - |
|  |  |  |  |  |  |  |  |

Abbreviations: MSE, mean squared error.

^a^Positive psychotic symptoms were assessed using the positive psychotic symptom rating on the Positive and Negative Syndrome Scale (Kay et al., 1987).

^b^Depressive symptoms were assessed using the Beck Depression Inventory (Beck et al., 1996).

^c^Not included in multivariate analysis because no symptom-locked activity at baseline.

**p* ≤ 0.05.

Improvement in depressive symptoms was best predicted by activation elicited by fearful and happy faces. A focal pattern of activation for fearful faces in the superior frontal gyrus as well as more widespread patterns of activation for happy faces across frontal, sensorimotor and midbrain regions contributed to the predictive model for depressive symptoms (Supplementary Table 5). Separate assessment of each affective condition revealed that improvement in depressive symptoms could be predicted by fearful faces (*r*=0.59, *p*=0.002) and happy faces (*r*=0.32, *p*=0.05), but not by neutral faces (Supplementary Table 4).

Supplementary Table 5. Predictors of improvement in depressive symptoms and their relative weights in predictive power (percentage of the total weights in the decision function); clusters < 10 active voxels excluded.

|  |  |  |  | | | | | |  |  | | | |  | | |
| --- | --- | --- | --- | --- | --- | --- | --- | --- | --- | --- | --- | --- | --- | --- | --- | --- |
| Anatomical region | | | | MNI coordinates | | |  | Brodmann area | | | | weight (%) | | | size (voxels) | |
|  | | | | x | y | z |  | | | |  | |  | | |  |
|  | | | |  |  |  |  | | | |  | |  | | |  |
| **Fearful faces (73.1%)** | | | |  |  |  |  | | | |  | |  | | |  |
| L superior frontal gyrus | | | | -10 | 28 | 52 | 8 | | | |  | | 74.1 | | | 20 |
|  | | | |  |  |  |  | | | |  | |  | | |  |
| **Happy faces (25.7%)** | | | |  |  |  |  | | | |  | |  | | |  |
| L inferior frontal gyrus | | | | -52 | 24 | 28 | 44 | | | |  | | 12.7 | | | 84 |
| R precentral gyrus | | | | 42 | 0 | 52 | 6 | | | |  | | 9.8 | | | 21 |
| L fusiform gyrus | | | | -40 | -38 | -20 | 37 | | | |  | | 5.8 | | | 62 |
| L insula | | | | -40 | -4 | -8 | 13 | | | |  | | 4.7 | | | 22 |
| L caudate | | | | -11 | 10 | -4 |  | | | |  | | 4.5 | | | 10 |
| L precentral gyrus | | | | -16 | -30 | 60 | 4 | | | |  | | 4.1 | | | 99 |
| R lingual gyrus | | | | 22 | -56 | -4 | 19 | | | |  | | 4.0 | | | 17 |
| R middle temporal gyrus | | | | 50 | -10 | -14 | 22 | | | |  | | 3.6 | | | 14 |
| L paracentral lobule | | | | -10 | -52 | 66 | 7 | | | |  | | 3.4 | | | 14 |
| L superior parietal gyrus | | | | -22 | -38 | 66 | 1 | | | |  | | 3.0 | | | 38 |
| R postcentral gyrus | | | | 20 | -36 | 64 | 1 | | | |  | | 2.9 | | | 21 |
| R precuneus | | | | 14 | -44 | 66 | 7 | | | |  | | 2.8 | | | 81 |
| R supplementary motor area | | | | 14 | 0 | 66 | 6 | | | |  | | 2.6 | | | 16 |
| R paracentral lobule | | | | 12 | -32 | 64 | 4 | | | |  | | 2.5 | | | 44 |
| R superior parietal gyrus | | | | 20 | -42 | 66 | 5 | | | |  | | 2.4 | | | 27 |
| L olfactory gyrus | | | | -31 | 3 | -15 | 34 | | | |  | | 1.8 | | | 21 |
| L superior frontal gyrus | | | | -14 | 31 | 59 | 8 | | | |  | | 1.4 | | | 10 |
|  | | | |  |  |  |  | | | |  | |  | | |  |

**Assessment of predictive models in independent treatment-as-usual group**

Changes in both positive psychotic and depressive symptoms could not be predicted using the multiple kernel learning model. Processing of ambiguous ‘neutral’ and fearful faces contributed most to the predictive model for positive psychotic symptoms (Supplementary Figure 1), whereas processing of fearful and happy faces contributed most to the predictive model for depressive symptoms (Supplementary Figure 2).

**
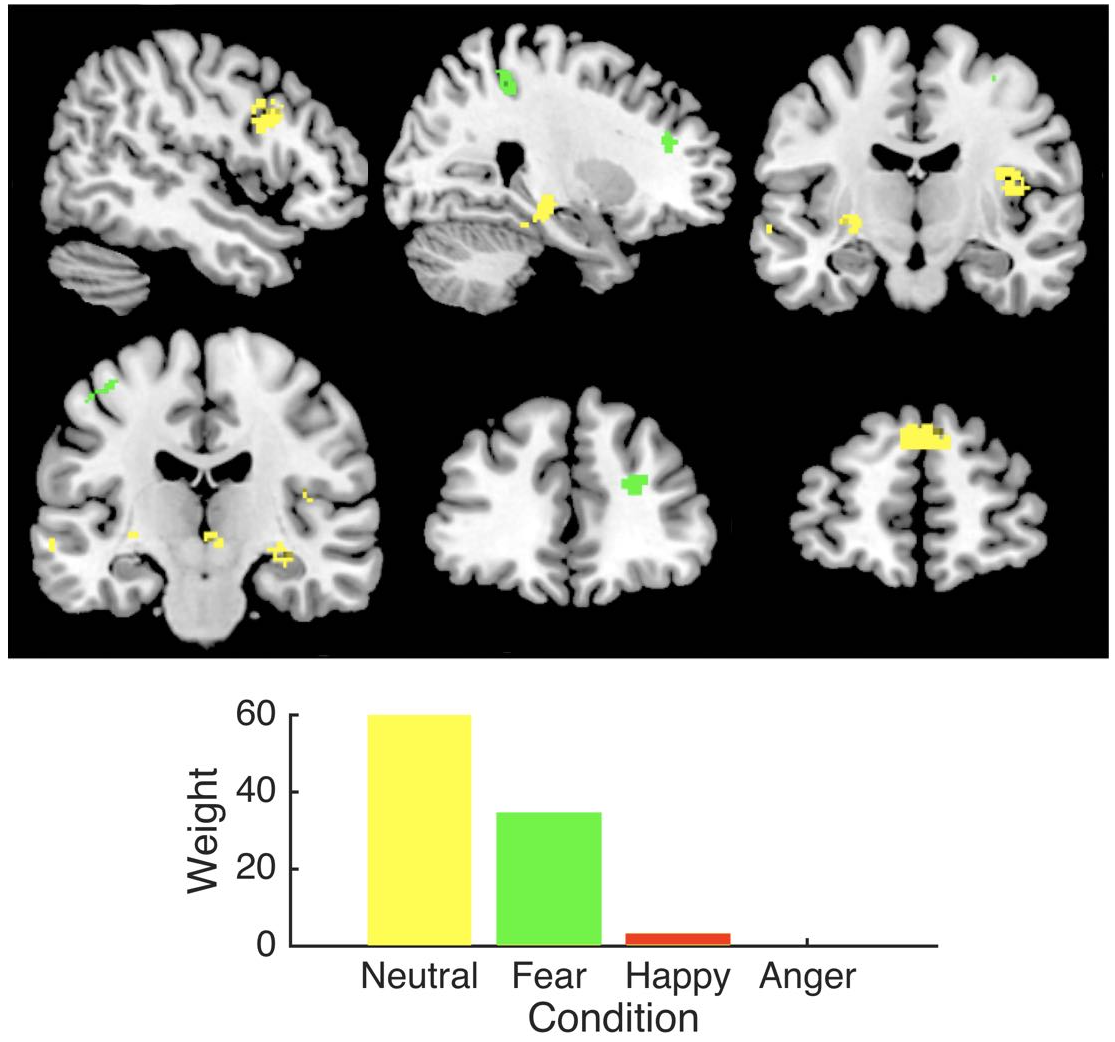
**

Supplementary Figure 1. Baseline brain responses to ambiguous ‘neutral’ and fearful faces do not predict changes in positive psychotic symptoms following treatment-as-usual. The multiple kernel learning model is not significant (*r*=-0.39, *p*=0.46). The bar graph shows the relative contribution of each condition to the decision function. Top, from left to right: x=48, 26, y=-12. Bottom, from left to right: y=-18, 39, 56.


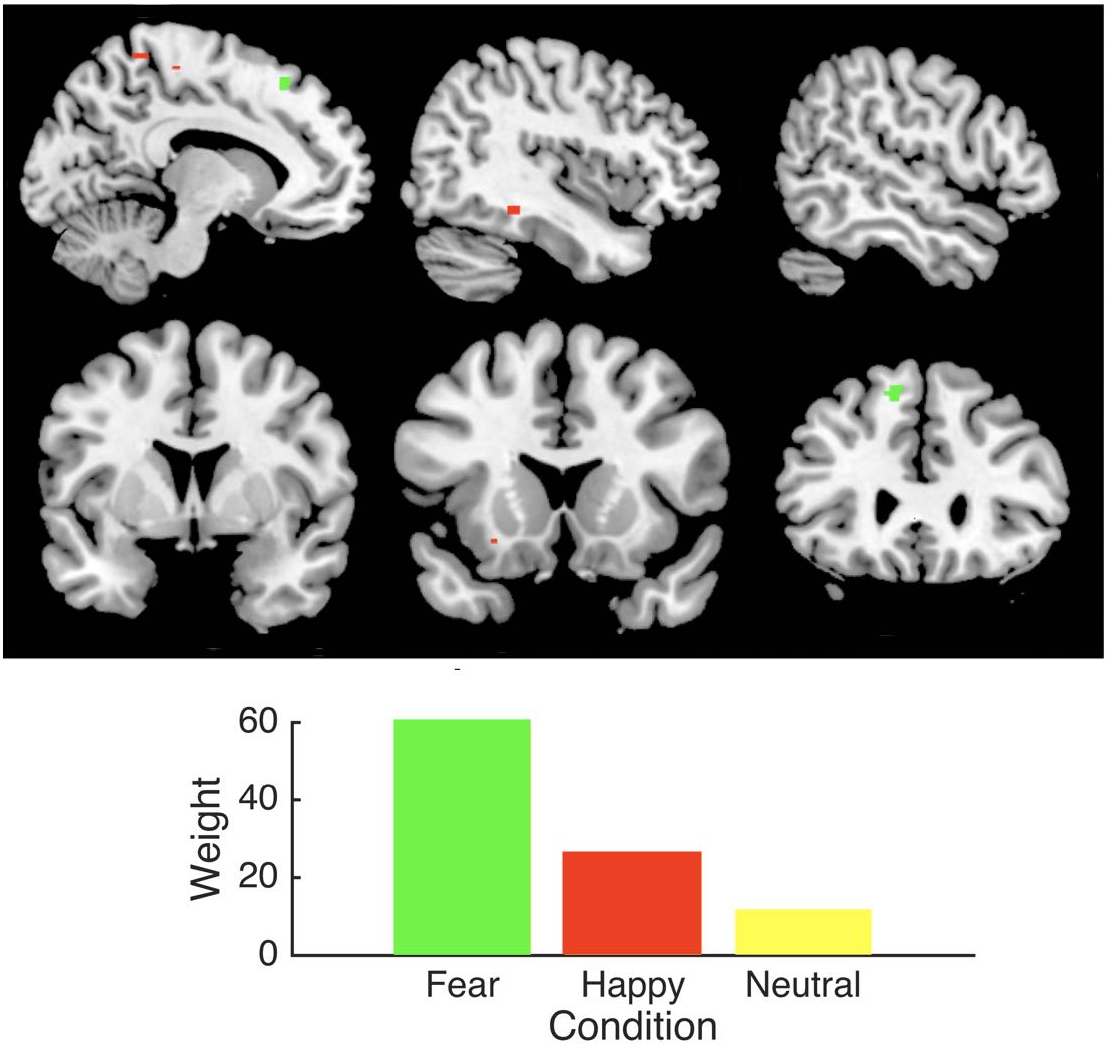


Supplementary Figure 2. Baseline brain responses to fearful and happy faces do not predict changes in depressive symptoms following CBTp. The multiple kernel learning model is not significant (*r*=-0.05, *p*=0.31). The bar graph shows the relative contribution of each condition to the decision function. Top, from left to right: x=-9, 41, 53. Bottom, from left to right: y=4, 17, 29.

**Whole brain analyses in CBTp and independent treatment-as-usual group**

Changes in symptoms following both CBTp and treatment-as-usual could not be predicted for both positive psychotic and depressive symptoms using the multiple kernel learning model (Supplementary Table 6, 7). Whilst the pattern was similar in terms of which affective conditions predicted changes in symptoms, with activation for ambiguous ‘neutral’ and fearful faces being the strongest predictor for positive psychotic symptoms and activation for happy and fearful faces being the strongest predictor for depressive symptoms, the overall model did not reach significance.

Supplementary Table 6. Predictive accuracy of whole-brain multivariate models for response to cognitive behavioural therapy for psychosis.

|  | *r* |  | *P*(*_r_*) |  | MSE |  | *P*_(MSE)_ |
| --- | --- | --- | --- | --- | --- | --- | --- |
|  |  |  |  |  |  |  |  |
| **Positive psychotic symptoms**^a^ | **-0.38** |  | **0.72** |  | **15.60** |  | **0.42** |
| Angry faces (30.0%) |  |  |  |  |  |  |  |
| Neutral faces (29.9%) |  |  |  |  |  |  |  |
| Fearful faces (24.8%) |  |  |  |  |  |  |  |
| Happy faces (15.3%) |  |  |  |  |  |  |  |
|  |  |  |  |  |  |  |  |
| **Depressive symptoms**^b^ | **-0.01** |  | **0.15** |  | **132.78** |  | **0.53** |
| Happy faces (80.2%) |  |  |  |  |  |  |  |
| Fearful faces (19.6%) |  |  |  |  |  |  |  |
| Neutral faces (0.3%) |  |  |  |  |  |  |  |
| Angry faces^c^ |  |  |  |  |  |  |  |
|  |  |  |  |  |  |  |  |

Abbreviations: MSE, mean squared error.

^a^Positive psychotic symptoms were assessed using the positive psychotic symptom rating on the Positive and Negative Syndrome Scale (Kay et al., 1987).

^b^Depressive symptoms were assessed using the Beck Depression Inventory (Beck et al., 1996).

^c^Not included in multivariate analysis because no symptom-locked activity at baseline.

Supplementary Table 7. Assessment of whole-brain predictive models in independent treatment-as-usual group.

|  | *r* |  | *P*(*_r_*) |  | MSE |  | *P*_(MSE)_ |
| --- | --- | --- | --- | --- | --- | --- | --- |
|  |  |  |  |  |  |  |  |
| **Positive psychotic symptoms**^a^ | **-0.11** |  | **0.18** |  | **18.33** |  | **0.26** |
| Fearful faces (62.6%) |  |  |  |  |  |  |  |
| Neutral faces (21.7%) |  |  |  |  |  |  |  |
| Angry faces (15.3%) |  |  |  |  |  |  |  |
| Happy faces (0.4%) |  |  |  |  |  |  |  |
|  |  |  |  |  |  |  |  |
| **Depressive symptoms**^b^ | **-0.31** |  | **0.65** |  | **47.13** |  | **0.29** |
| Happy faces (60.0%) |  |  |  |  |  |  |  |
| Fearful faces (33.6%) |  |  |  |  |  |  |  |
| Neutral faces (6.4%) |  |  |  |  |  |  |  |
| Angry faces^c^ |  |  |  |  |  |  |  |
|  |  |  |  |  |  |  |  |

Abbreviations: MSE, mean squared error.

^a^Positive psychotic symptoms were assessed using the positive psychotic symptom rating on the Positive and Negative Syndrome Scale (Kay et al., 1987).

^b^Depressive symptoms were assessed using the Beck Depression Inventory (Beck et al., 1996).

^c^Not included in multivariate analysis because no symptom-locked activity at baseline.

**References**

Beck A., Steer R., Brown G., Manual for the Beck Depression Inventory-II, 1996, Psychological Corporation; San Antonia, TX.

Chu C., Ni Y., Tan G., et al., Kernel regression for fMRI pattern prediction. *Neuroimage* 2010; 56: 662-673.

Kay S., Fiszbein A., Opler L., The Positive and Negative Syndrome Scale for schizophrenia. *Schizophr. Bull.* 1987; 13: 261-276.

Schrouff J., Rosa M.J., Rondina J.M., et al., PRoNTo: Pattern recognition for neuroimaging toolbox. *Neuroinformatics* 2013; 11: 319-337.

Schrouff J., Monteiro J., Rosa M., et al., Can we interpret linear kernel machine learning models using anatomically labelled regions? In: *Organ. Hum. Brain Mapp.* 2014.

Shawe-Taylor J., Cristianini N., *Kernel Methods for Pattern Analysis*. Cambridge University Press; 2004.

Svetnik V., Liaw A., Tong C., Variable selection in random forest with application to quantitative structure-activity relationship. *Proceedings of the 7^th^ Course on Ensemble Methods for Learning Machines* 2004; 1-8.
